# Nutritional Approach to Cancer Cachexia: A Proposal for Dietitians

**DOI:** 10.3390/nu14020345

**Published:** 2022-01-14

**Authors:** Kotone Tanaka, Sho Nakamura, Hiroto Narimatsu

**Affiliations:** 1 School of Nutrition and Dietetics, Faculty of Health and Social Services, Kanagawa University of Human Services 1-10-1 Heiseicho, Yokosuka-shi 238-0013, Japan; 2 Cancer Prevention and Control Division, Kanagawa Cancer Center Research Institute 2-3-2 Nakao, Asahi-ku, Yokohama 241-8515, Japan; research@nakasho.org (S.N.); hiroto-narimatsu@umin.org (H.N.); 3 Graduate School of Health Innovation, Kanagawa University of Human Services, 3-25-10 Research Gate Building 2-A, Tonomachi, Kawasaki-ku, Kawasaki 210-0821, Japan; 4 Department of Genetic Medicine, Kanagawa Cancer Center, 2-3-2 Nakao, Asahi-ku, Yokohama 241-8515, Japan

**Keywords:** cancer, cachexia, EPA, HMB, creatine, carnitine

## Abstract

Cachexia is one of the most common, related factors of malnutrition in cancer patients. Cancer cachexia is a multifactorial syndrome characterized by persistent loss of skeletal muscle mass and fat mass, resulting in irreversible and progressive functional impairment. The skeletal muscle loss cannot be reversed by conventional nutritional support, and a combination of anti-inflammatory agents and other nutrients is recommended. In this review, we reviewed the effects of nutrients that are expected to combat muscle loss caused by cancer cachexia (eicosapentaenoic acid, β-hydroxy-β-methylbutyrate, creatine, and carnitine) to propose nutritional approaches that can be taken at present. Current evidence is based on the intake of nutrients as supplements; however, the long-term and continuous intake of nutrients as food has the potential to be useful for the body. Therefore, in addition to conventional nutritional support, we believe that it is important for the dietitian to work with the clinical team to first fully assess the patient’s condition and then to safely incorporate nutrients that are expected to have specific functions for cancer cachexia from foods and supplements.

## 1. Introduction

Cachexia is a common, related factor of malnutrition in patients with cancer [1]. Cancer cachexia is a multifactorial syndrome defined by an ongoing loss of skeletal muscle mass (with or without loss of fat mass) that leads to progressive functional impairment [2]. The agreed-upon diagnostic criteria for cachexia are weight loss of 5% or more or weight loss of 2% or more in a person who is already depleted according to current weight and height (body mass index [BMI] less than 20 kg/m^2^) or loss of skeletal muscle mass (sarcopenia) [2].

Cancer cachexia is caused by a complex combination of anorexia, increased protein catabolism, systemic inflammation, and increased resting energy expenditure. Its pathophysiology is characterized by a negative balance of protein and energy caused by various combinations of reduced food intake and metabolic abnormalities. The main features of cachexia are a strong tendency toward catabolism and a negative protein–energy balance that is difficult to restore [2,3]. As a result, total body skeletal muscle mass is irreversibly reduced, leading to decreased Activities of Daily Living (ADL), worse prognosis, and decreased quality of life (QOL) [4,5,6]. Therefore, in supporting cancer patients, nutritional interventions should be a multimodal approach in conjunction with exercise and pharmacotherapy to address muscle mass loss. In addition, nutritional management for cancer cachexia should focus on preventing the loss of muscle mass based on nutritional risk assessment and evaluation [7]. Moreover, cancer cachexia gradually worsens as cancer progresses and can be divided into three main stages: pre-cachexia, cachexia, and refractory cachexia [2]. It is not uncommon for cancer patients to have limited food intake due to anorexia caused by adverse events of treatment or worsening of their general condition [8,9]; in the refractory cachexia stage, aggressive nutritional support is not recommended [10]. Therefore, early nutritional intervention is important for cachexia.

However, it has been reported that conventional nutritional supplements, such as energy and protein supplementation alone, do not improve cancer cachexia, and nutritional therapy, such as anti-inflammatory nutrients, should be used in combination [11,12]. In fact, energy and protein supplementation alone have been reported to improve weight in cancer patients with cachexia [11,13,14,15,16,17] or subjective outcomes such as QOL [13]. However, many reports have found no impact on secondary outcomes (e.g., improved ADLs and reduced mortality) [11,13,14,15,16,17], with few improvement reports [18]. Therefore, conventional nutritional management should be implemented in the future, such as energy and protein supplementation, as well as a new nutritional approach to counter cancer cachexia [18].

While the American Society of Clinical Oncology (ASCO) Guideline [19] and the European Society for Oncology (ESMO) Clinical Practice Guidelines [20] state that the importance of dietary nutrient intake is emphasized, the evidence for the intake of certain nutrients, such as n3 fatty acids, is considered insufficient due to lack of data and low quality. However, these are investigations of intake as supplements, not evidence of long-term intake of nutrients as a diet. Moreover, even if we focus on the effects of a particular nutrient, the interaction with other nutrients may result in more favorable effects from dietary intake than from supplements [21].

The purpose of this study was to review the knowledge of specific nutrient intake as an approach that can be taken by dietitians in clinical practice among the wide range of approaches to cancer cachexia and to propose an approach that can be taken at present.

## 2. Selected Articles for This Review

For this review, we searched at PubMed (MEDLINE) to identify clinical studies published from January 1992 to August 2021 that evaluated the effects of each nutrient in cancer patients with cachexia. Searches were conducted using search terms including “cancer” and “cachexia”. Study reports were retrieved regardless of whether these were full publications or abstracts. The studies that evaluated a single nutrient were included in the review; those that evaluated combinations of multiple nutrients were not included.

Eligible criteria of this review are as follows. First, randomized, controlled trials (RCTs) that were double blind, single blind, or unblinded and clinical trials were included. Second, Trials of patients with a confirmed diagnosis of advanced cancer and a clinical judgement of cachexia or related conditions (independent of gender, age, or race) were included. Third, studies with the following primary and secondary outcomes measures were reviewed in the study. The primary outcome measures included weight gain, body composition, and nutritional status and the secondary outcome measures assessed included side effects and adverse events.

As a result of the literature search, 127 papers on EPA, 10 papers on HMB, 75 papers on creatine, and 21 papers on carnitine were retrieved. Of these, four papers on EPA, two papers on HMB, two papers on creatine, and two papers on carnitine that met the eligible criteria and included our research questions were selected as the target papers for this review.

## 3. Nutritional Interventions to Support Cancer Cachexia Management

### 3.1. Eicosapentaenoic Acid (EPA)

#### 3.1.1. General Information

EPA is an unsaturated fatty acid that cannot be synthesized by the body [22]. EPA reduces the production of inflammatory cytokines such as IL-6 and TNF-α and decreases the activity of proteolysis-inducing factors (PIF) [23,24]. The recommended daily intake of EPA is 2.0 g/day for men and 1.6 g/day for women [22] between the ages of 30 and 49 years. Fish oil containing high levels of EPA and DHA is prone to developing a fishy or metallic taste [25]. The side effects associated with EPA overdose include gastrointestinal symptoms, liver dysfunction, and bleeding tendency [26].

#### 3.1.2. Mechanisms of EPA on Cancer Cachexia

Cancer cells produce pro-inflammatory cytokines such as IL-6 and TNF-α, which produce inflammatory reactions between cancer cells and their hosts. They also secrete a PIF, which increases muscle catabolism. These functions are related to the effects of EPA. Therefore, in recent years, EPA intake has been considered to regulate these cancerous inflammations and bring metabolism back from a hypercatabolic state to a normal state [23,24].

#### 3.1.3. Clinical Evidence of EPA

##### Effect of EPA on Cancer Cachexia

The effect of EPA in cancer cachexia patients is currently unknown. Studies in patients with advanced pancreatic cancer [27,28] or in lung cancer patients undergoing chemotherapy [29] reported that EPA intake resulted in an increase in body weight or LBM. Conversely, in a study of advanced cancer patients, EPA intake did not improve nutritional status or function [30]. Three studies [27,28,29] found increases in muscle mass and ADLs, suggesting that EPA may be effective in preventing muscle loss and increasing muscle mass in cancer cachexia patients.

##### EPA Intake

The recommended intake of EPA for cancer cachexia patients is about 2 g/day. The studies that reported EPA-induced increases in body weight or muscle mass [27,29,31] generally took between 1.8 and 2.2 g. In interpreting this result, we need to consider the side effects associated with EPA overdose [26]. According to a statement by the European Food Safety Authority, the upper limit of EPA intake is 1.8 g, even when taken as a medicine. However, it has been reported that, in general, there are few side effects from excessive intake of EPA [32], although there have been studies that have shown nausea and loss of appetite in some subjects after taking up to 6 g of EPA [28]. Therefore, it should not be considered overly dangerous, but its use in patients with a tendency to bleed from tumors should be avoided.

##### The Length of Time It Takes for the Effects of EPA to Appear

It takes about a month for EPA to be effective in muscle protection in cancer cachexia patients. The rationale is that studies reported that the studies that were found to be effective had intervention periods of 8–16 weeks [27,28,29], and a study reported that weight gain was observed up to week 4, after which weight was maintained [28]. The studies that found no effect were short interventions of 2 weeks [30]. Therefore, it would be better to continue EPA supplementation for more than 4 weeks for cancer cachexia.

#### 3.1.4. Clinical Recommendation

Theoretically, EPA may have an effect on cancer cachexia by preventing muscle catabolism. However, there is no unified view. As long as it is taken appropriately, the possibility of serious side effects is considered to be low. To increase the number of studies and obtain accurate evidence, it is advisable to actively take nutrients safely. Currently, when EPA is to be ingested, the amount of EPA should be about 2 g/day, and the period until the effect appears should be set at approximately 4 to 12 weeks; it is necessary to evaluate the effect. EPA is reduced by overheating, especially in fried foods, where it is reduced the most [33]. Since blue fish such as sardines, tuna, and mackerel contain high amounts of EPA [22], it is best to eat these fish raw to get the most from their diet.

### 3.2. β-Hydroxy-β-Methylbutyrate (HMB)

#### 3.2.1. General Information

HMB is a leucine metabolite, one of the essential amino acids. It has been reported that approximately 5% of leucine is converted to HMB in the body [34]. Although HMB is believed to promote protein anabolism and inhibit protein catabolism in muscle [35,36], its mechanism is not clearly understood [37]. However, HMB has been reported to affect muscle hypertrophy in healthy young men [38,39]. It has also been reported that bedridden elderly who received HMB through tube feeding received a protective effect on muscle protein catabolism [40]. The most common way to consume HMB is in the form of HMB calcium salt, and the recommended intake of HMB alone is 3 g (3000 mg)/day [37]. Regarding the side effects associated with HMB overdose, studies in humans and animals have not shown any adverse effects associated with HMB supplementation [37].

#### 3.2.2. Mechanisms of HMB on Cancer Cachexia

Cancer-induced cachexia accelerates protein catabolism and decreases protein anabolism, resulting in a shortened life expectancy and reduced ADL and QOL. Although the effects of HMB are not clear [36], it has been suggested that HMB appears to attenuate phosphorylation of p42/44-mitogen-activated protein kinase by PIF [41]. Therefore, it is possible that HMB promotes muscle protein anabolism and inhibits muscle protein catabolism [41].

#### 3.2.3. Clinical Evidence of HMB

##### Effect of HMB on Cancer Cachexia

The effect of HMB in cancer cachexia patients is currently unknown. A study in which HMB, arginine, and glutamine were administered to advanced cancer patients reported that FFM and weight gain were observed [42], while there were reports of no significant increase in LBM [43]. Therefore, although the protective effect of HMB on muscle proteins in cancer cachexia patients is unknown, it may be expected to be effective. In interpreting the results, it is important to note that all of the studies included 3 g of HMB, 14 g of arginine, and 14 g of glutamine, and none of the studies revealed cancer cachexia with HMB alone. However, although the effects of arginine and glutamine on cancer cachexia have been reported [44], the effects of HMB alone on rats and mice assuming a cancer cachexia state have also been widely reported [45]. Therefore, HMB alone may have a protective effect on muscle proteins against cancer cachexia. To use a dietary supplement containing HMB similar to the composition of the above study, it should be considered using an amino acid beverage containing HMB, L-arginine, and L-glutamine.

##### HMB Intake

The recommended intake of HMB for cancer cachexia patients is expected to be approximately 3 g/day. A study of cancer cachexia patients receiving 3 g of HMB, 14 g of arginine, and 14 g of glutamine reported a significant increase in LBM and FFM [42,43]. Conversely, there are no reports on the maximum tolerable dose for HMB. A study of healthy subjects reported that ingestion of 6 g of HMB did not result in further muscle hypertrophy, but no symptoms were associated with overdose [38]. Therefore, whether there is a new role for muscle protein protection when cancer cachexia patients consume more than 3 g of HMB is unknown, although the risk of adverse events is thought to be low.

##### The Length of Time It Takes for the Effects of HMB to Appear

It takes about 4 weeks for HMB to be effective in muscle protection in cancer cachexia patients. The rationale is that studies performed for 8 weeks tended to have a higher LBM throughout the study period [43], and studies performed for 24 weeks had weight gain 4 weeks after the beginning of HMB intake, which was then maintained for 24 weeks [42]. In both studies, the effects tended to manifest themselves in the form of physical changes between 4 and 8 weeks. Therefore, it would be better to continue HMB supplementation for more than 4 weeks for cancer cachexia.

#### 3.2.4. Clinical Recommendation

Theoretically, HMB may have a protective effect on muscle proteins against cancer cachexia. However, there is no unified view. As long as it is taken appropriately, the possibility of serious side effects is considered to be low. To increase the number of studies and obtain accurate evidence, it is advisable to actively take nutrients safely. Currently, when HMB is to be ingested, the amount of HMB should be approximately 3 g/day and the period until the effect appears should be set at more than 4 weeks; it is necessary to evaluate the effect. Leucine is found in animal proteins, including meat such as beef, loin ham, and liver; seafood such as horse mackerel, salmon, and bonito flakes; dairy products such as cheese and skimmed milk powder; and soy products such as dried tofu [46]. Furthermore, since leucine is an essential amino acid, it is necessary to consume leucine and other essential amino acids in sufficient quantities. Therefore, to obtain HMB from the diet, animal protein should be actively consumed.

### 3.3. Creatine

#### 3.3.1. General Information

Creatine is synthesized daily by the liver and kidneys from 1 to 2 g of three amino acids: glycine, arginine, and methionine. Approximately 95% of synthesized creatine is included in skeletal muscle and is used primarily as an energy source for muscles [47]. The effects of creatine on muscle mass and strength have been reported in many studies, especially in young individuals [48,49]. It has been reported that the effect is ambiguous on the elderly [50,51] and no adverse events were reported [51]. Although there is no clear daily dose of creatine, it is recommended to take the most common program that involves an initial loading phase of 20 g/day for 5–7 days, followed by a maintenance phase of 3–5 g/day for different periods of time (1 week to 6 months) [52]. It has been reported that a long-term intake of 3 g/day can have the same effect as loading [53].

#### 3.3.2. Mechanisms of Creatine on Cancer Cachexia

The proposed mechanisms underlying the beneficial effects of carnitine to prevent skeletal muscle loss by cancer cachexia are based on stabilization of mitochondrial mem-brane by lipids’/phospholipids’ synthesis and reduction in the amount of free long-chain fatty acids and preserving the activities of key mitochondrial enzymes in energy metabolism, oxidative phosphorylation, and also anti-inflammatory and anti-oxidative properties [54,55]. Therefore, in recent years, creatine intake has been considered to have a protective effect on muscles by resisting the catabolic states of cancerous muscle protein catabolic states [51,54,56].

#### 3.3.3. Clinical Evidence of Creatine

##### Effect of Creatine on Cancer Cachexia

The effect of creatine in patients with cancer cachexia is currently unknown. Studies of patients with colorectal cancer undergoing chemotherapy or with cancer cachexia have shown no changes in muscle mass or body composition [57,58]. Conversely, studies in rats reported that it inhibited tumor growth and metastasis in cancer-bearing rats [59] and suppressed weight loss and skeletal muscle atrophy [60]. Thus, although creatine may influence cancer cachexia, there is no evidence for this. The reason creatine had no effect on the cancer cachexia pathology in the previous study might be its mechanism of action. Regarding its effect on cancer patients, it has been reported that it is beneficial in maintaining and increasing somatic cell mass in patients receiving less invasive chemotherapy, while it may not be beneficial in patients receiving more invasive chemotherapy [55]. Therefore, we believe that cancer cachexia patients may benefit from taking creatine in the early stages of cancer development when their metabolic status is relatively similar to that of healthy individuals. Moreover, some points should be noted in applying the results of these studies. In general, the main short-term side effects of creatine intake are decreased kidney function and fluid retention. However, current systematic reviews have ruled out both impaired renal function and water retention as side effects of excessive creatine intake [61,62]. Furthermore, reviews of the effects of creatine on cancer cachexia have reported no serious adverse events [51]. However, care should be taken to avoid adverse effects when creatine is taken by the elderly, people with renal disease, people using diuretics, people whose renal function is expected to be impaired by chemotherapy, and people with ascites or edema due to advanced cancer.

##### Creatine Intake

Current evidence suggests that creatine intake in cancer cachexia patients is ineffective; but if taken, the recommended creatine intake is about 3g/day. The rationale is that two studies in cancer cachexia patients [57,63] found no effect; but, in healthy subjects, 20 g of creatine for 6 days or 3 g of creatine for about 28 days was reported to be effective [53]. As a caveat in interpreting the results, the study by Jatoi et al. [58] used the study by Hultman et al. [53] as a reference to determine creatine intake. Nonetheless, cancer cachexia patients did not show the same effects as healthy indexes. This suggests that metabolic abnormalities in patients with cancer cachexia may prevent them from benefiting from creatine. Therefore, taking creatine before cachexia or in the early stages of cachexia may be effective.

##### The Length of Time It Takes for the Effects of Creatine to Appear

If creatine effectively prevents muscle catabolism in cancer cachexia patients, it will take about a month to observe its effects. The rationale is that these studies in healthy individuals reported positive effects after 6 weeks [48] and 14 weeks [50] of intervention, and creatine levels in body tissues were completed in about 28 days [53]. However, it is not clear if the same is true for cancer cachexia patients.

#### 3.3.4. Clinical Recommendation

Theoretically, creatine does not have a muscle protective effect against cancer cachexia, according to current evidence. However, there is no unified view. As long as it is taken appropriately, the possibility of serious side effects is considered to be low. To increase the number of studies and obtain accurate evidence, it is advisable to actively take nutrients safely. Currently, when creatine is to be ingested, the amount of creatine should be about 3 g/day, the period until the effect appears should be set at more than 4 weeks, and it is necessary to evaluate the effect. Creatine is found in fish, such as herring, salmon, and tuna, and meat, such as pork and beef [64]. Therefore, to obtain creatine from the diet, animal protein should be actively consumed.

### 3.4. Carnitine

#### 3.4.1. General Information

Carnitine comprises two amino acids, lysine and methionine; most of the carnitine in the body (95%) is stored in the skeletal muscle [65]. In skeletal muscle, carnitine plays an important role in increasing fat oxidation while conserving glycogen, delaying fatigue during prolonged aerobic exercise [66]. Carnitine can be synthesized endogenously or obtained exogenously from the diet, especially from red meat [65]. Therefore, deficiencies are usually rare, and the Food and Nutrition Board (FNB) of the National Academies has not established a recommended Dietary Reference Intakes (DRI) for carnitine per day [67]. However, many cancer cachexia patients are carnitine deficient [68,69]. This has been attributed to decreased dietary intake due to the multifactorial etiology of cachexia, impaired endogenous synthesis [70], increased urinary excretion due to chemotherapy [71], and decreased skeletal muscle [65]. Therefore, carnitine deficiency has been proposed to be an underlying cause of cancer cachexia [72] and tumor-associated fatigue [71].

#### 3.4.2. Mechanisms of Carnitine on Cancer Cachexia

In recent years, it has been suggested that carnitine may help cancer patients to maintain and increase muscle mass and improve fatigue by antagonizing their hypercatabolic metabolism [73], playing a predominant role in the generation of cancer cachexia [74].

#### 3.4.3. Clinical Evidence of Carnitine

##### Effect of Carnitine on Cancer Cachexia

The effect of carnitine in cancer cachexia patients is currently unknown. The rationale is that in a study of advanced pancreatic cancer patients and advanced tumor patients, the results showed a significant increase in BMI, and improved nutritional status (body cell mass, body fat) [75] or nutritional variables (lean body mass and appetite) were significantly increased [73]. There have been no reports of serious side effects or worsening of the condition due to carnitine intake. For this study, the effect of carnitine supplementation on cancer cachexia was based mainly on the assumption that the patient is carnitine deficient. However, in the study by Cruciani et al. [76], only 30% of the patients were carnitine deficient. Therefore, in actual clinical practice, it is advisable to test patients for carnitine deficiency before starting carnitine supplementation, considering the possibility that cancer cachexia patients may not necessarily be carnitine deficient.

##### Carnitine Intake

The recommended intake of carnitine in cancer cachexia patients is approximately 3 g/day. The rationale is that studies have shown that an effect in preventing muscle catabolism has been achieved by taking 4–6 g of carnitine [73,77]. There was no significant difference from the placebo group in the study of 2 g of carnitine per day [78]. A different study by the same researchers reported that carnitine may be safely administered at doses of up to 3000 mg/day [76]. On the other hand, carnitine supplements have been reported to cause side effects such as nausea, vomiting, abdominal cramps, and diarrhea when administered [79]. In contrast, the highest dose of carnitine administered in this study was 6 g, but no adverse events occurred. Therefore, the carnitine intake should be set at 3 g, and the dosage should be maintained.

##### The Length of Time It Takes for the Effects of Carnitine to Appear

It takes about 4 weeks for carnitine to be effective in muscle protection in cancer cachexia patients. The rationale is that studies reported that the studies that were found to be effective had intervention periods of 12 weeks [75] and 4 weeks [73]. Moreover, a study that also used a 4-week intervention showed that carnitine plasma levels were reported to increase significantly [78]. Therefore, it would be better to continue carnitine supplementation for more than 4 weeks for cancer cachexia.

#### 3.4.4. Clinical Recommendation

Theoretically, carnitine may have a protective effect on cancer cachexia by preventing muscle catabolism. However, there is no unified view. As long as it is taken appropriately, the possibility of serious side effects is considered to be low. To increase the number of studies and obtain accurate evidence, it is advisable to actively try to take nutrients safely. Currently, when carnitine is to be ingested, the amount of carnitine should be about 3 g/day, the period until the effect appears should be set at approximately 4 weeks, and it is necessary to evaluate the effect. Carnitine is abundant in animal foods such as red meat, fish meat, poultry, and milk [79]. Usually, the redder the color of the meat, the higher the carnitine content. Therefore, to obtain carnitine from the diet, red-colored meat, such as beef, should be actively consumed.

### 3.5. Combination of Nutrients

The summary table for the nutrients discussed in this review is shown in Table 1. There is no evidence that the nutrients discussed in this review, when taken as supplements, effectively prevent muscle hypercatabolism due to cancer cachexia. However, as reviewed in this paper, the nutrients themselves may benefit cancer patients with cachexia. Furthermore, side effects are extremely rare when taken in the amounts and for the periods recommended in this review. To increase the number of studies, we believe it is important to first practice it safely in a clinical setting. The guidelines for cancer cachexia list salmon, a nutritious food, as a natural source of omega-3 fats [19]; therefore, the idea of taking each nutrient in the diet is shown in Table 2. Consistent, long-term intake of nutrients as a diet may be useful for the human body. Besides the effects of nutrients, the intake of tasty diets can contribute to the improvement of QOL due to the pleasure of eating. Therefore, it is recommended that these foods be consciously consumed during the early stages of cancer development.

## 4. Discussion

### 4.1. Multi-Nutrient Combinations

Interventions that combine multiple nutrients may be more effective than single nutrient supplementation. When EPA and the amino acids leucine, arginine, and methionine were used together, the amount of protein synthesis almost doubled [80]. Alternatively, in the fish oil study, supplementation of the diet with the all-in combination of high protein, leucine, and fish oil significantly reduced carcass loss, muscle, and fat mass, and improved muscle performance. Furthermore, the total daily activity normalized after intervention with a specific nutritional combination [81]. In a study of mice fed a macronutrient containing carnitine and mice fed an intervention diet showed a higher cumulative food intake compared to controls. In addition, the intervention group had a significantly lower tumor weight and no metastases [59]. However, there is still little evidence on the intake of multiple nutrients.

As in Section 3.5, with a nutritional approach that aims to replenish a certain nutrient when deficient, would give benefit with a single nutrient supplement. However, when administering nutrients for their effect on cancer cachexia, multiple nutrient intakes are likely to be more useful than a single intake, provided they are not burdensome to the patient. In this context, the nutritional approach should first assess the patient’s condition. As in the past, if energy, protein, and other nutrients are lacking, they need to be supplemented. In addition, we propose that the new nutritional approach should consider cancer cachexia and supplement nutrients simultaneously. For example, if a patient is eating well and maintaining his or her weight, a nutrient approach such as creatine may be a good choice to increase muscle mass. If the patient is eating well but losing weight, a multi-nutrient approach may be more effective in preventing the progression of cancer cachexia. Alternatively, for patients who cannot eat and lose weight, a conventional nutritional approach may be important first, supplementing with nutrients to the extent possible within the amount of food they can ingest.

Furthermore, many of the nutrients introduced in this review are contained in blue fish and red meat. Therefore, actively consuming blue fish and red meat is not only a general nutritional supplement for protein but also a nutrient supplement to counteract muscle loss due to cancer cachexia. From the perspective of providing more nutrients with less stress, it is important to consider that food supplements should also be more efficient than taking a large number of supplements at once.

### 4.2. Time of Nutrient Supplementation

Cancer cachexia is a condition that gradually worsens as cancer progresses. Fearon et al. described three stages of cachexia syndrome: pre-cachexia, cachexia, and refractory cachexia [2]. In the pre-cachexia stage, there are relatively few symptoms of cachexia and few metabolic abnormalities, while, in the refractory cachexia stage, active management is no longer possible [2]. Therefore, it is important to initiate interventions earlier in the management of cachexia to control or delay its progression. The above nutritional interventions must be performed while the progression of cancer cachexia is at an earlier stage. As for creatine in particular, it is suggested that it may become less effective as the state of muscle anabolism and catabolism as a result of cancer cachexia progresses, as mentioned earlier. Since it is difficult to slow the progression of cachexia by conventional nutritional management alone [2], it is necessary to begin interventions such as nutrient addition early in nutritional interventions for patients to maintain their nutritional status.

Aggressive administration of nutrition to refractory cachexia is discouraged [4]. In fact, in patients with refractory cachexia, oral nutrition has been reported to have harmed not prolonged life [77]. The National Comprehensive Cancer Network Guidelines for Palliative Care recommend that enteral and parenteral nutrition be considered (as needed) only if the prognosis is longer than a few weeks to a few days [82]. In the terminal stage, not only in cancer treatment, the goal shifts from treatment to care and the patient’s QOL should be the most important consideration. The nutritional approach with the expectation of improvement in nutritional status is considered to be at least from the pre-cachexia to cachexia stages.

The nutrients discussed in this study should be administered simultaneously with conventional energy and protein supplementation. Therefore, the patient’s condition should be evaluated (assessed) by a dietitian who is in charge of adjusting the entire diet. Moreover, each nutrient should be added after explaining the level of evidence to the patient at the same time as the diet is adjusted. To increase the level of evidence for nutritional approaches, more research is needed on the addition of nutrients to conventional nutritional management. Since sufficient evidence is needed to make nutritional approaches more effective and safer for patients, it is necessary to take an active nutritional approach and gather evidence with full consideration of the risks.

### 4.3. Case-Oriented Examples

In this review, we introduce nutrient-oriented examples. In this section, we discuss case-oriented examples that would benefit actual clinical practice.

Case 1: A man with early-stage cancer, preoperatively for radical surgery. Since postoperative weight (muscle) loss was expected, nutritional guidance was provided before the surgery. Currently, his appetite is normal, with no weight loss. The patient had no nutritional problems or inflammatory reactions. His compliance is good. It is expected that this patient will be able to eat normally, approximately 3 months after the surgery.

In such cases, it is expected that the effects of increased muscle catabolism due to cancer cachexia have not yet occurred. Since there is no significant inflammatory response, it is recommended to manage the patient’s nutrition to increase muscle mass as much as possible. It is recommended that the daily protein requirements should be based on animal proteins. It is also recommended to add ham sandwiches to meals as snacks. In addition, muscle training along with protein intake can help increase muscle mass as much as possible before surgery and prepare for weight loss after surgery. It is important to remember that the goal of the patient’s treatment is to achieve a cure and that good nutritional management in the present situation will affect the patient’s recovery after surgery.

Case 2: A woman with metastatic cancer undergoing palliative chemotherapy. The patient has been losing weight rapidly over the past year and is still progressing. Nutritional guidance was provided to prevent weight loss. The patient’s physical strength decreased along with weight loss, and she also had anorexia. The patient had chronic high levels of inflammatory response. Both the patient and her family had a strong sense of crisis and were worried about what she should eat. However, due to chemotherapy-induced taste disorder and nausea, she could not eat as much as she would have liked.

In such cases, the inflammatory response is high, and the catabolism of muscles due to cancer cachexia is advanced; thus, it is not easy to increase muscle mass even with protein intake and exercise. Therefore, it is advisable to first determine what foods the patient can eat and how much of them she can eat, and then recommend the consumption of blue fish and other foods that contain anti-inflammatory nutrients such as EPA. When a patient has anorexia or taste disorder, cold or sour foods may be easier to eat. In this case, a small amount of sushi or marinated fish may be effective. It is important to note that the purpose of the patient’s treatment is shifting from cure to care; it is not necessary to force the patient to eat but it is necessary to provide guidance within the scope of what the patient can currently eat.

### 4.4. Limitation

One limitation of this study is that the types of cancers in the cited literature were not examined in detail. Due to the small number of studies, some nutrients were only examined in the cachexia of biased cancer types and the results may have been influenced by those cancer types. Furthermore, we could not quantify lean body mass and muscle mass due to different measurement methods and indices. Molecular or functional elucidation of the improvement of lean body mass by nutritional supplementation should be studied in the future. The findings of this study have such limitations to apply for cancer patients; therefore, it is recommended that appropriate nutrient intake should be reviewed by the patient’s medical team when practiced in clinical practice.

## 5. Conclusions

Cancer cachexia is particularly common in patients with cancer and is associated with various symptoms that decrease the patient’s QOL, such as loss of appetite and muscle mass. It is conceivable that the nutrients discussed in this review may be effective in preventing muscle hypermetabolism due to cancer cachexia. We believe that it is important for the dietitian to work with the clinical team to assess the patient’s condition, symptoms, QOL, and wishes, and then to safely incorporate nutrients that are expected to have specific functions for cancer cachexia.

## Figures and Tables

**Table 1:** Summary table.

Nutrients	Author and Year	Patients		Intervention	Duration of Intervention	Results
		Total no. of Patients	Patient Characteristics			
EPA	Fearon (2003) [27]	*n* = 200	Patients with unresectable pancreatic cancer	620 kcal, protein 32 g +/- EPA 2.2 g/day	8 weeks	Weight ↑ LBM ↓
	Wigmore (2000) [28]	*n* = 26	Patients with advanced pancreatic cancer	EPA starting at 1 g/day, increased to 6 g/day over four weeks followed by 6 g/day	12 weeks	Weight ↑
	Murphy (2011) [29]	*n* = 40	Patients with nonsmall cell lung cancer	EPA 2.2 g/day	95 ± 3.8 days	Weight →
	Bruera (2003) [30]	*n* = 60	Patients with advanced cancer	EPA 1.8 g and DHA 1.2 g/day or placebo	14 days	No significant difference in nutritional status and fanction
HMB	May(2002) [42]	*n* = 49	Patients with solid tumors who had demonstrated a weight loss of at least 5%	HMB 3 g, arginine 14 g and glutamine 14 g/day or an isocaloric control mixture of nonessential amino acids	4 weeks	Weight ↑
	Berk (2008) [43]	*n* = 472	Patients with advanced cancer weight loss of 2% to 10%	HMB 6 g, arginine 28 g and glutamine 28 g/day or an isocaloric control mixture of equal nitrogen	8 weeks	No significant difference in LBM
Creatine	Norman (2006) [57]	*n* = 30	Patients with colorectal cancer undergoing chemotherapy	Creatine 45 g/day load×1 week and was then reduced to 22.5 g/day	8 weeks	BCM ↑( patients undergoing less aggressive chemotherapy )
	Jatoi (2017) [58]	*n* = 263	Patients with incurable malignancy other than a primary brain tumor	Creatine 20 g/day load×5 days followed by 2 g/day or placebo	4 weeks	No significant difference in body composition
Carnitine	Gramignano (2006) [73]	*n* = 12	Patients with advanced cancer	L-Carnitine 6 g/day	4 weeks	LBM ↑
	Kraft (2012) [75]	*n* = 72	Patients with advanced pancreatic cancer	L-Carnitine 4 g/day or placebo	12 weeks	BMI ↑

Abbreviations: EPA, Eicosapentaenoic acid; HMB, β-hydroxy-β-methylbutyrate; LBM, lean body mass; BCM, body cell mass; ↑,Increase; ↓, Decrease; →, Maintain.

**Table 2:** List of proposed nutrients.

	EPA	HMB	Creatine	Carnitine	Combined Nutrition
Foods with high content	blue fish such as sardines, tuna, and mackerel	(leucine) beef, loin ham, liver, horse mackerel, salmon, bonito flakes, cheese, skimmed milk powder, dried tofu	fish such as herring, salmon, and tuna, and in meat such as pork and beef	red meat, fish meat, poultry, and milk	
Menu (something easy to eat when patients have a poor appetite)	sushi and sashimi of sardines, tuna, and mackerel	beef bowl, beef stew, ham sandwich, sushi, dishes with horse mackerel and salmon, grilled salmon, cheese, cream stew, stewed dried tofu	dishes made with herring, salmon, tuna, pork shabu salad, beef rice bowl, beef stew	pork shabu salad, beef bowl, fish dish, beef stew, milk, cream stew	sushi and sashimi platters, seafood rice bowls, nabe dishes, sandwiches, cream stew
Total recommended daily consumption	2 g/day	3 g/day	3 g/day	3 g/day	
Corresponding literature	[23,24]	[38,39,40]	[51,54,56]	[73,74]	

Abbreviations: EPA, Eicosapentaenoic acid; HMB, β-hydroxy-β-methylbutyrate.

## Data Availability

Not applicable.
